# Enhanced nanoscale resistive switching memory characteristics and switching mechanism using high-Ge-content Ge_0.5_Se_0.5_ solid electrolyte

**DOI:** 10.1186/1556-276X-7-614

**Published:** 2012-11-06

**Authors:** Sheikh Ziaur Rahaman, Siddheswar Maikap, Atanu Das, Amit Prakash, Ya Hsuan Wu, Chao-Sung Lai, Ta-Chang Tien, Wei-Su Chen, Heng-Yuan Lee, Frederick T Chen, Ming-Jinn Tsai, Liann-Be Chang

**Affiliations:** 1 Department of Electronic Engineering, Chang Gung University, 259 Wen-Hwa 1st Road, Kwei-Shan, Tao-Yuan, 333, Taiwan; 2 Material and Chemical Research Laboratories, Industrial Technology Research Institute, Hsinchu, 310, Taiwan; 3 Electronic and Opto-Electronic Research Laboratories, Industrial Technology Research Institute, Hsinchu, 310, Taiwan

**Keywords:** nanoscale, memory, resistive switches, high Ge, solid electrolyte

## Abstract

We demonstrate enhanced repeatable nanoscale bipolar resistive switching memory characteristics in Al/Cu/Ge_0.5_Se_0.5_/W, as compared with Al/Cu/Ge_0.2_Se_0.8_/W structures, including stable AC endurance (>10^5^ cycles), larger average SET voltage (approximately 0.6 V), excellent data retention (>10^5^ s) at 85°C, and a high resistance ratio (>10^4^) with a current compliance of 8 μA and a small operation voltage of ±1.5 V. A small device size of 150 × 150 nm^2^ and a Cu nanofilament with a small diameter of 30 nm are both observed by high-resolution transmission electron microscope in the SET state. The Ge_*x*_Se_1 − *x*_ solid electrolyte compositions are confirmed by both energy-dispersive X-ray spectroscopy and X-ray photoelectron spectroscopy. The switching mechanism relies on the smaller barrier heights for holes rather than for electrons; the positively charged Cu^*z*+^ ions (i.e., holes) migrate through the defects in the Ge_*x*_Se_1 − *x*_ solid electrolytes during SET/RESET operations. Hence, the Cu nanofilament starts to grow at the Ge_0.5_Se_0.5_/W interface, and starts to dissolve at the Cu/Ge_0.5_Se_0.5_ interface, as illustrated in the energy band diagrams. Owing to both the higher barrier for hole injection at the Cu/Ge_0.5_Se_0.5_ interface than at the Cu/Ge_0.2_Se_0.8_ interface and greater thermal stability, the resistive switching memory characteristics of the Al/Cu/Ge_0.5_Se_0.5_/W are improved relative to the Al/Cu/Ge_0.2_Se_0.8_/W devices. The Al/Cu/Ge_0.5_Se_0.5_/W memory device can also be operated with a low current compliance of 1 nA, and hence, a low SET/RESET power of 0.61 nW/6.4 pW is achieved. In addition, a large memory size of 1,300 Pbit/in^2^ is achieved with a small nanofilament diameter of 0.25 Å for a small current compliance of 1 nA.

## Background

Resistive switching random access memory (RRAM) devices have recently become promising candidates for future low-power nanoscale nonvolatile memory applications [1-3]. RRAM devices involving materials such as HfO_*x*_[4,5], SrTiO_3_[6], TiO_2_[7,8], ZrO_2_[9,10], Na_0.5_Bi_0.5_TiO_3_[11], NiO_*x*_[12,13], ZnO [14], TaO_*x*_[15,16], and AlO_*x*_[17,18] are widely reported. However, their precise switching mechanism remains unclear, despite being important for applications. On the other hand, other resistive switching memory materials exploit the migration of cations (Ag^+^ or Cu^*z*+^, *z* = 1 and 2) in solid electrolytes such as Ge_*x*_Se_1 − *x*_[19-21], GeS_2_[22], Ta_2_O_5_[23], SiO_2_[24], Ag_2_S [25,26], ZrO_2_[27], TiO_*x*_/ZrO_2_[28], GeSe_*x*_/TaO_*x*_[29], HfO_2_[30], CuTe/Al_2_O_3_[31], Ti/TaO_*x*_[32], and GeO_*x*_[33]. Resistive switching memory that uses Cu/ZnO/Pt [34], Ag/SiO_2_/Pt [35], and Ag/ZrO_2_/Pt [36] structures has also been reported recently. Further, recent studies also conclude that the growth of a metallic filament, which is at the heart of the conduction mechanism, is initiated at the Cu/ZnO (or Ag/SiO_2_ or Ag/ZrO_2_) interface and that its dissolution starts at the ZnO/Pt (or SiO_2_/Pt or ZrO_2_/Pt) interface, in contrast to previously reported results. Therefore, a better understanding of the switching mechanism based on the formation and dissolution of the Cu or Ag filament in solid electrolytes is required for future applications. In this regard, the Ge_*x*_Se_1 − *x*_ (*x* = 0.2 to 0.4) solid electrolytes have attracted considerable interest. In these, mobile Cu^*z*+^ or Ag^+^ ions play an important role in the formation and dissolution of the metallic filament [19-21]. Furthermore, important benefits of using Ge_*x*_Se_1 − *x*_ as switching materials are their 100% device yield and their ease of processing. Kund et al. [37] reported GeSe-based resistive switching memory in an Ag/GeSe/W structure with a current compliance (CC) of 10 μA and showing data retention up to 70°C. Jeong et al. [38] reported threshold switching using Pt/GeSe/Pt structures. Although Se-rich Ge_0.3_Se_0.7_ (or Ge_0.2_Se_0.8_) solid electrolytes have been extensively studied [19-21,29,37], there are no reports on solid electrolytes with a low Se (or, equivalently, a high Ge) content, such as Ge_0.5_Se_0.5_, showing enhanced memory performance. The melting points of Se and Ge are 220.5°C and 937.4°C, respectively, suggesting that the thermal stability and the memory characteristics can both be improved by increasing the relative Ge content. In this study, we investigated an Al/Cu/Ge_0.5_Se_0.5_/W memory device with improved resistive switching memory characteristics compared to those of an Al/Cu/Ge_0.2_Se_0.8_/W device. They include the repeatability of switching cycles (>10^3^), a larger SET voltage (*V*_SET_) of approximately 0.6 V (due to the greater barrier height for holes or Cu^*z*+^ ions), stability, and long AC endurance (>10^5^ cycles) when operated with a small voltage of ±1.5 V. The composition of the solid electrolytes was confirmed by energy-dispersive X-ray spectroscopy (EDX) and X-ray photoelectron spectroscopy (XPS). The barrier height for hole injection at the Cu/Ge_0.5_Se_0.5_ interface (0.75 eV) is lower than that for electron injection at the Ge_0.5_Se_0.5_/W interface (0.91 eV), so hole injection dominates. The positively charged Cu^*z*+^ ions (i.e., holes) migrate and start to grow at the Ge_0.5_Se_0.5_/W interface and then start to dissolve at the Cu/Ge_0.5_Se_0.5_ interface. We investigated this process in terms of energy band diagrams. The barrier height for hole injection at the Cu/Ge_0.5_Se_0.5_ interface is also greater than at the Cu/Ge_0.2_Se_0.8_ interface (0.75 vs. 0 eV). This affects the migration of Cu^*z*+^ ions via defects as well as the formation and dissolution of Cu filaments in the SET and RESET operations in the Cu/Ge_0.5_Se_0.5_ solid electrolyte. Furthermore, we observe better stability in data retention in both the high-resistance state (HRS) and the low-resistance state (LRS) (over >10^5^ s) at 85°C in the Al/Cu/Ge_0.5_Se_0.5_/W memory device compared to the Al/Cu/Ge_0.5_Se_0.5_/W device. This results from the better thermal stability of the Ge_0.5_Se_0.5_ switching material. A Cu nanofilament diameter of 30 nm is also observed by high-resolution transmission electron microscopy (HRTEM) under SET conditions in the 150 × 150 nm^2^ memory device. The Al/Cu/Ge_0.5_Se_0.5_/W memory device can be operated with a low CC of 1 nA, an appropriate value for future atomic-scale devices on the scale of 0.25 Å.

## Methods

**Figure 1:**
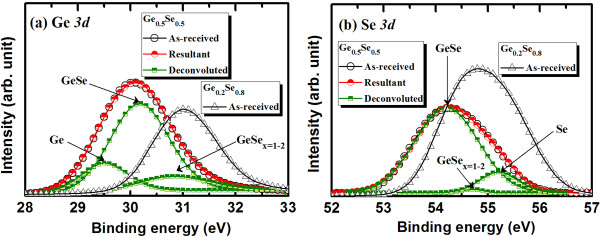
**XPS spectra.** (**a**) Ge 3*d* and (**b**) Se 3*d* core-level electrons for Ge_0.5_Se_0.5_and Ge_0.2_Se_0.8_solid electrolytes.

Eight-inch-diameter Si (100) wafers were first cleaned using the standard Radio Corporation of America process, and an approximately 200-nm-thick SiO_2_ layer was deposited onto the wafers. A bottom electrode (BE) made of W or TiN metal was then deposited onto the SiO_2_/Si substrates. The BE thickness was approximately 100 nm. To design the memory devices, a SiO_2_ layer of thickness approximately 150 nm was deposited on the upper surface and developed by optical lithography over an active area of 150 × 150 nm^2^. A Ge_0.5_Se_0.5_ film with a nominal thickness of 40 nm was then deposited onto the active regions using an electron gun and pure Ge_0.5_Se_0.5_ granules. The chamber vacuum was 5 × 10^−6^ Torr prior to deposition. A slow deposition rate of approximately 2 Å/s helped to control the deposited thickness precisely. A Cu metal layer of nominal thickness 40 nm was deposited using a thermal evaporator, to serve as a top electrode (TE). A 160-nm-thick layer of Al was then deposited, using the same thermal evaporator, to protect the Cu surface from oxidation at high temperatures. The total TE thickness (Cu + Al) was approximately 200 nm. We investigated the resistive switching mechanism by comparing different TEs, made of Al, W, or IrO_*x*_. Finally, a lift-off process produced the resistive switching memory devices in an Al/Cu/Ge_0.5_Se_0.5_/W structure (device type, S1). We compared them with similar devices made using an Al/Cu/Ge_0.2_Se_0.8_/W structure (device type, S2). The Ge_0.5_Se_0.5_ and Ge_0.2_Se_0.8_ materials were characterized by XPS. After deposition, the Ge_0.5_Se_0.5_/W (or Ge_0.2_Se_0.8_/W) sample was transferred immediately to the analyzing XPS vacuum chamber (1 × 10^−9^ Torr). A film of nominal thickness 10 nm was etched out from the surface before taking the spectra. The analysis area had a diameter of 650 μm. All spectra were calibrated using a reference C 1*s* peak at 284.6 eV. Figure 1a shows the XPS spectra of Ge 3*d* core-level electrons. In the case of the Ge_0.5_Se_0.5_/W samples, the peak binding energy of Ge 3*d* core-level electrons was 30.1 eV, higher than that of the pure Ge 3*d* peak (29 eV) [39]. This suggests that the Ge 3*d* peak centered at 30.1 eV represents the GeSe composition. The GeSe_*x*_ spectrum shows a GeSe peak centered at 30.2 eV and a GeSe_2_ peak centered at 30.8 eV. The GeSe_*x*_ peak has a higher binding energy owing to the higher binding energy of Se 3*d* core-level electrons. Consequently, the binding energy of the Ge_0.2_Se_0.8_ films increases to 31.1 eV. Figure 1b shows the XPS spectra of the Se 3*d* core-level electrons. For the Ge_0.5_Se_0.5_/W sample, the peak binding energy of Se 3*d* electrons is 54.2 eV, lower than the Se 3*d* peak (55.2 eV). The peak binding energy of GeSe_2_ is 54.7 eV, and the corresponding binding energy of Se 3*d* core-level electrons in the Ge_0.2_Se_0.8_ film is approximately 54.8 eV. However, the chemical shift of Se 3*d* (55.5 eV) electrons is −1 eV for GeSe_2_[39]. The increase in binding energy with increasing Se content allows us to confirm the higher Se content in the GeSe_*x*_ film [39,40]. 

The thicknesses of the resistive switching material and of the memory device were evaluated from a HRTEM image. HRTEM was carried out using a FEI Tecnai G2 F-20 field-emission system (FEI Co., Hillsboro, OR, USA) with an operating voltage of 200 kV and a resolution of 0.17 nm. A molybdenum (Mo) grid was used for TEM observations. Memory characteristics, such as current–voltage (*I*-*V*) relations, endurance, and data retention were measured using an HP4156C semiconductor parameter analyzer (Agilent Technologies Inc., Santa Clara, CA, USA). Charge-trapping phenomena were observed by capacitance-voltage (*C*-*V*) measurements using the HP4284A LCR meter (Agilent Technologies Inc.). The frequency applied during the *C*-*V* measurement was 1 MHz. The capacitance was measured in parallel capacitance-conductance mode. For electrical measurements, the bias was applied to the TE while the BE was grounded. More than 100 devices were measured at random to assess the uniformity of the memory characteristics.

## Results and discussion

**Figure 2:**
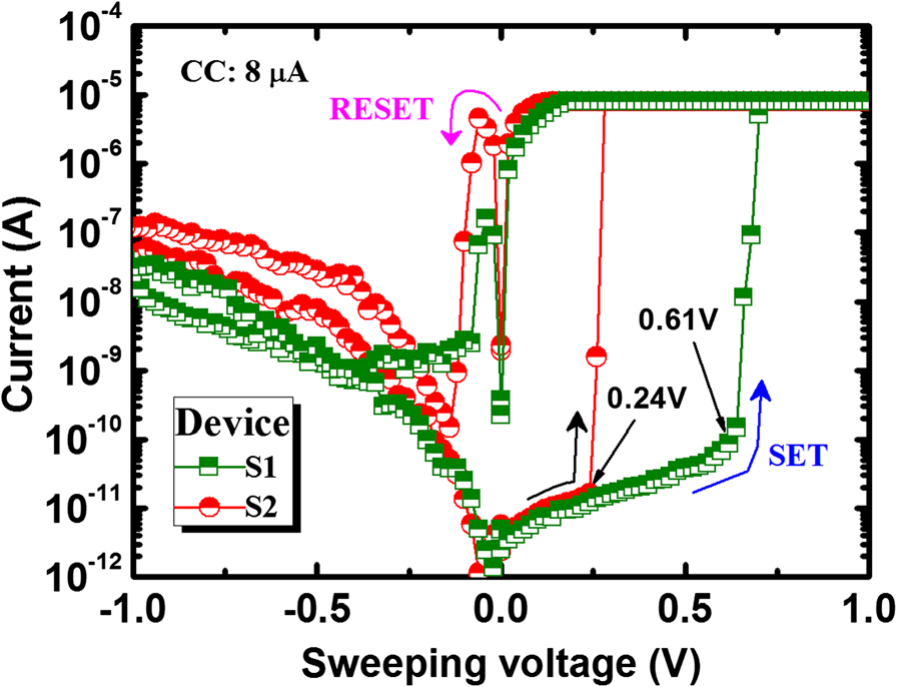
**Typical*I*-*V*hysteresis of the Al/Cu/Ge**_**0**.**2**_**Se**_**0**.**8**_**/W and Al/Cu/Ge**_**0**.**5**_**Se**_**0**.**5**_**/W memory devices.**

**Figure 3:**
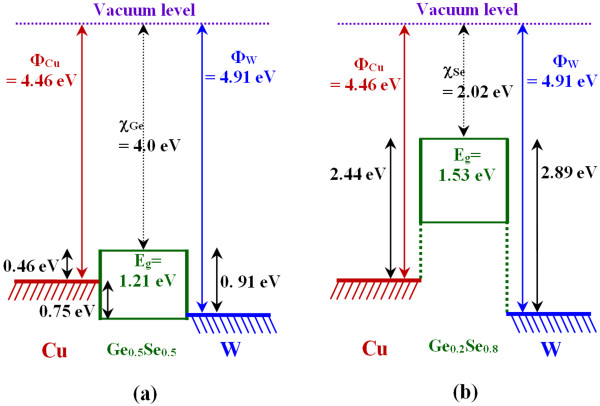
**Schematic energy band diagrams of** (**a**) **Al**/**Cu**/**Ge**_**0**.**5**_**Se**_**0**.**5**_/**W and** (**b**) **Al**/**Cu**/**Ge**_**0**.**2**_**Se**_**0**.**8**_/**W structures.**

**Figure 4:**
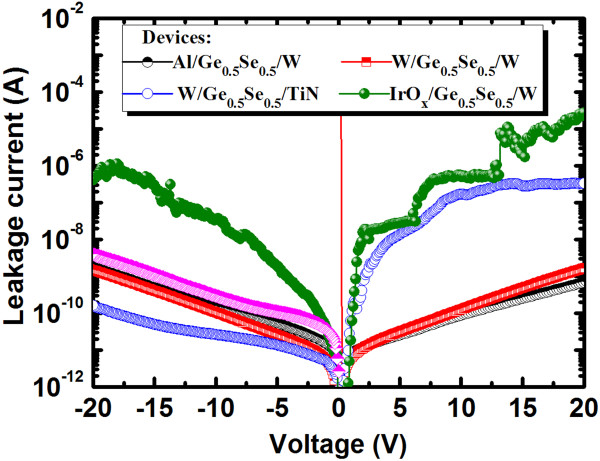
**Leakage currents for different electrodes made of Al, W, TiN, and IrO**_***x***_**.**

Figure 2 shows the typical bipolar resistive switching memory characteristics of Al/Cu/Ge_0.5_Se_0.5_/W and Al/Cu/Ge_0.2_Se_0.8_/W structures. Initially, the formation voltage is not necessary for these memory devices. Voltages are swept as follows: 0 → +1.3 V → 0→ −1.2 V → 0. The step voltage and the hold/delay time were 20 mV and 0.1 ms, respectively. A low CC of 8 μA and a small operation voltage of ±1.3V were applied. The leakage currents for the S1 and S2 devices at +0.3 V are lower (approximately 10 and 13 pA) than the leakage currents (approximately 100 and 180 pA) at −0.3 V because the work function (*Φ*_*m*_) of the W BE (approximately 4.91 eV [41]) is greater than that of the Cu TE (approximately 4.46 ± 0.3 eV [42]). These leakage currents are dominated by hole injection at the Cu/Ge_*x*_Se_1 − *x*_ interface rather than by electron injection at the Ge_*x*_Se_1 − *x*_/W interface, as explained in the schematic energy band diagrams below. The energy gap (*E*_g_) of the Ge_*x*_Se_1 − *x*_ films can be calculated from Vagard’s law [43]:

(1) $$E_{g}\left(Ge_{x}Se_{1-x}\right)=xE_{g}\left(Ge\right)+\left(1-x\right)E_{g}\left(Se\right)-bx\left(1-x\right)$$

where *b* is the bowing parameter, assumed to be zero here for simplicity. The energy gaps of Ge and Se are 0.67 and 1.74 eV, respectively [44]. Equation 1 gives *E*_*g*_ approximately 1.21 and 1.53 eV for the Ge_0.5_Se_0.5_ and Ge_0.2_Se_0.8_ films, respectively. Assuming electron affinities *χ*_Ge_ = 4.0 eV and *χ*_Se_ = 2.02 eV for Ge in the Ge_0.5_Se_0.5_ and Se in the Ge_0.2_Se_0.8_ films, respectively, the corresponding energy band diagrams of the S1 and S2 structures are shown in Figure 3. With a positive bias on the TE (0 < +*V* <*V*_SET_), the barrier height (0.75 eV) for hole injection at the Cu/Ge_0.5_Se_0.5_ interface is lower than that for electron injection at the Ge_0.5_Se_0.5_/W interface (0.91 eV, Figure 3a). With a negative bias on the TE (after RESET), the barrier height (0.3 eV) for hole injection at the W/Ge_0.5_Se_0.5_ interface is lower than that for electron injection (0.46 eV) at the Cu/Ge_0.5_Se_0.5_ interface in the Cu/Ge_0.5_Se_0.5_/W structures. In the Cu/Ge_0.2_Se_0.8_/W structures, the barrier height for hole injection is approximately zero compared to that for electron injection (Figure 3b). Therefore, the observed leakage currents are due to hole, rather than electron, injection. We also investigated the leakage currents using different electrodes, such as Al, TiN, and IrO_*x*_, in Ge_0.5_Se_0.5_ film, with results shown in Figure 4. The leakage currents for the Al/Ge_0.5_Se_0.5_/W, W/Ge_0.5_Se_0.5_/W, W/Ge_0.5_Se_0.5_/TiN, and IrO_*x*_/Ge_0.5_Se_0.5_/W devices were approximately 11.8, 11.2, 764, and 1,550 pA at +2 V, respectively, and approximately 22.4, 10.1, 6.7, and 173 pA at −2 V, respectively. The differences in leakage currents can be explained by considering the differences in the work functions of the metal electrodes (*Φ*_TiN_ = 4.42 eV [44], *Φ*_Al_ = 4.28 eV [45], and *Φ*_IrO*x*_ = 5 eV [46]). For the W/Ge_0.5_Se_0.5_/W devices, the leakage currents at both polarities are nearly the same (11.2 and 10.1 pA) because they share the same W electrode. Due to the lower *Φ*_*m*_ of the TiN BE, the leakage current is greater at +2 V than at −2 V for the W/Ge_0.5_Se_0.5_/TiN and IrO_*x*_/Ge_0.5_Se_0.5_/W devices (764 > 6.7 pA and 1,550 > 173 pA, respectively). The leakage current for the IrO_*x*_ electrode is greater than for the Al, TiN, and W electrodes (173 > 22.4, 10.1, and 6.7 pA) because of the inertness of IrO_*x*_ and Ir metal. On the other hand, the physical thickness of the switching material can be increased because of the reactivity of the Al, TiN, and W TEs during the deposition process. The leakage currents in all devices were measured by sweeping the voltage over ±20 V. However, resistive switching characteristics were not observed. On the other hand, all devices involving Cu electrodes show formation-free bipolar resistive switching characteristics (Figure 2), implying a 100% yield for this Al/Cu/Ge_0.5_Se_0.5_/W device. This suggests that the Cu electrode plays a key role in achieving such a good resistive switching behavior, as explained below. 

**Figure 5:**
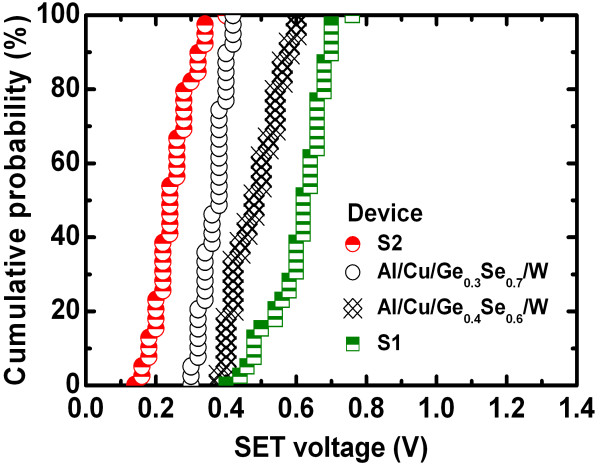
Cumulative probability of SET voltages for the S1 and S2 devices.

**Figure 6:**
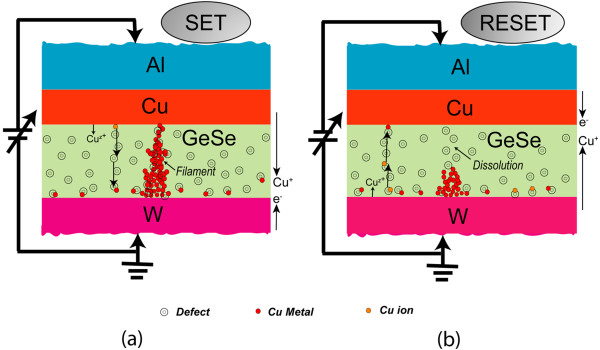
**Schematic views of the filament** (**a**) **formation and** (**b**) **dissolution under SET and RESET operations.**

**Figure 7:**
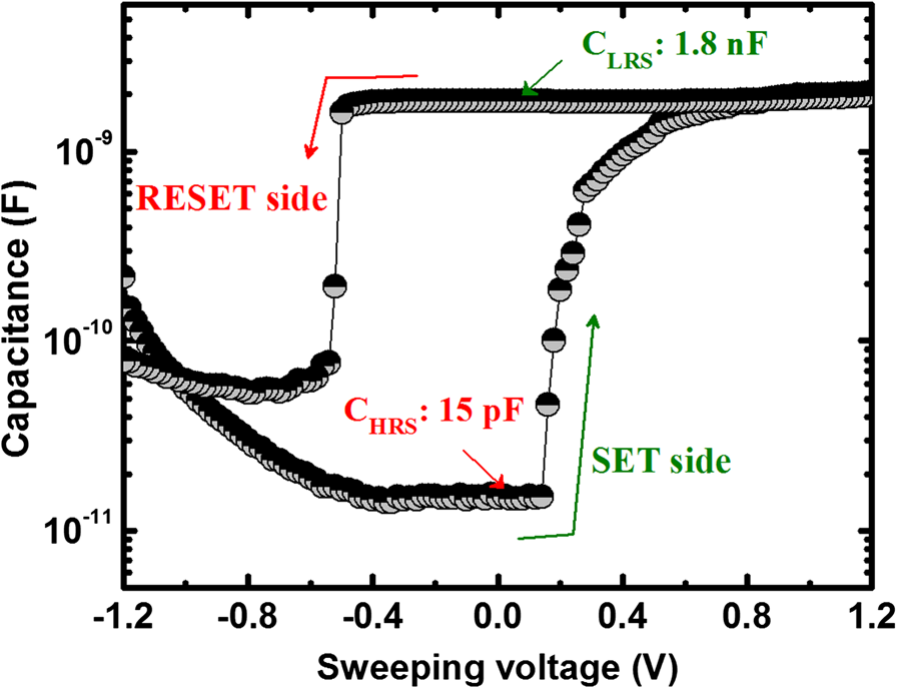
**Charge**-**trapping phenomena.** Observed by *C*-*V* measurement, proving the migration of Cu^*z*+^ions via defects in the Ge_*x*_Se_1 − *x*_ solid electrolytes.

Average values (standard deviation (SD)) of *V*_SET_ for the S1 and S2 devices were 0.61 V (0.08 V) and 0.24 V (0.06 V), respectively (Figure 5). In addition, the average values (SD) of *V*_SET_ for the Cu/Ge_0.3_Se_0.7_/W and Cu/Ge_0.4_Se_0.6_/W devices were also 0.36 V (0.04 V) and 0.48 V (0.07 V), respectively. The average *V*_SET_ increases with increasing Ge content in the Ge_*x*_Se_1 − *x*_ solid electrolyte because the lower concentration of defects hinders the migration of Cu^*z*+^ ions and also because the barrier height for holes at the Cu/Ge_0.5_Se_0.5_ interface (0.75 eV) is greater than at the Cu/Ge_0.2_Se_0.8_ interface (0 eV). In essence, GeSe represents an archetypal chalcogenide glass-forming system, whereas Ge and Se atoms are predominantly four- and two-coordinated, respectively [47,48]. However, Se is one of the most important semiconductors and has an unusual crystal structure, consisting of chains and rings of two-coordinated Se atoms. The addition of Ge creates Ge-Se heteropolar bonds that constitute GeSe_4_ tetrahedra [47,49]. According to the phase-separated model, the stoichiometric glass consists of Se-rich and Ge-rich clusters. However, a Se-rich cluster is visualized as a two-chain-wide fragment of the layer structure of *α*-GeSe_2_ bordered by Se-Se bonds, whereas a Ge-rich cluster consists of ethane-like units [48]. The presence of Se-rich clusters in the glass produces a less compact structure with a significant concentration of distorted tetrahedra. Considering both the floppy-type glass and the energy band diagram of the Ge_0.2_Se_0.8_ film, the average *V*_SET_ of <0.24 V is small, as reported by several researchers [19,21,37]. Due to the absence of a barrier for hole injection at the Cu/Ge_0.2_Se_0.8_ interface and in floppy-type glass, uncontrolled Cu migration can occur even at low *V*_SET_ and with a low CC of 1 nA (discussed later). A lower *V*_SET_ is undesirable for memory circuit design because it narrows the read margin and can cause error. However, the larger *V*_SET_ of 0.61 V obtained for the Al/Cu/Ge_0.5_Se_0.5_/W structure, a value of great interest for applications, has not been reported to date. Under SET conditions (+*V* >*V*_SET_), the HRS switches to the LRS through the formation of a Cu metallic nanofilament in the solid electrolyte (Figure 2). In this case, the positively charged Cu^*z*+^ ions (or holes) migrate toward the W BE and take electrons from the W BE, resulting in the growth of a conical Cu nanofilament from the W BE in the Ge_*x*_Se_1 − *x*_ solid electrolyte through electrochemical reduction (Cu^*z*+^ + *ze*^–^ → Cu, Figure 6a). Due to the smaller barrier height for holes, Cu^*z*+^ ions, rather than electrons, migrate. Consequently, the device sets in a LRS. Under RESET conditions (−*V* <*V*_RESET_), the LRS switches back to the HRS through the dissolution of the Cu metallic filament at the Cu/Ge_*x*_Se_1 − *x*_ interface, by electrochemical oxidation (Cu → Cu^*z*+^ + *ze*^–^, Figure 6b). This results from the higher electric field at this interface, caused by the high resistance at the neck of the conical Cu nanofilament. The kinetics of filament formation in these Ge_*x*_Se_1 − *x*_ solid electrolytes is different from oxide-based materials reported elsewhere [34-36]. We note that the Cu^*z*+^ ions can migrate through porous regions in the solid electrolyte or via defects. Therefore, defective solid electrolytes or high-κ materials can be used to produce such a resistive switching behavior. We performed *CV* measurements on a defective solid electrolyte. Figure 7 shows typical *CV* hysteresis characteristics when sweeping voltages as −1.5 V → +1 V → −1.5V. The capacitances in the HRS (*C*_HRS_) and LRS (*C*_LRS_) are approximately 15 pF and 1.8 nF, respectively, at a read voltage of 0.06 V. *CV* hysteresis is caused by charge trapping, likely due to Cu^*z*+^ ions moving through the defect sites or being trapped in the Ge_*x*_Se_1 − *x*_ solid electrolyte. 

**Figure 8:**
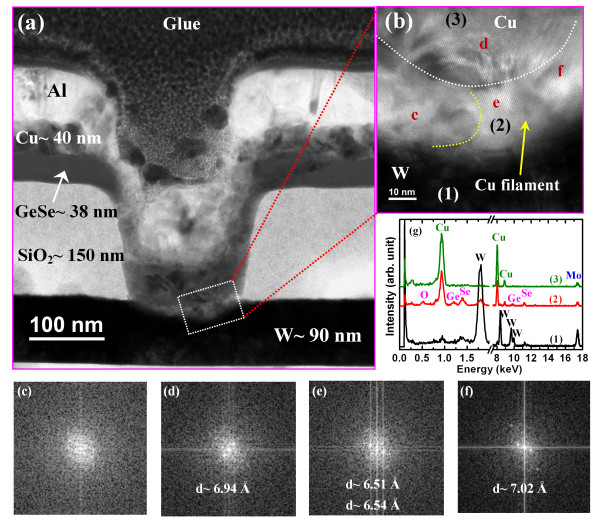
**Cu**^***z*+**^** ion migration and filament formation.** (**a**) HRTEM image of an Al/Cu/Ge_0.2_Se_0.8_/W resistive memory device after 6 × 10^3^ P/E cycles and the device kept in SET condition. (**b**) The diameter of the Cu filament is approximately 30 nm. (**c**) Amorphous Ge_0.2_Se_0.8_ film is observed at the without-filament region by FFT. (**d**) FFT in Cu electrode. (**e**) to (**f**) Crystalline Cu filament is observed. (**g**) EDX analysis at the points indicated in (b). The numbers (1), (2), and (3) indicated in (b) correspond to the EDX analysis positions.

To investigate Cu nanofilament formation, we prepared a typical Al/Cu/Ge_0.2_Se_0.8_/W memory device for TEM observation. Before performing the TEM measurement, the memory device was characterized with 6,000 program/erase (P/E) cycles. Finally, the memory device was kept in the SET condition. The P/E current and pulse width were 500 μA and 500 μs, respectively. Cu^*z*+^ ion migration and filament formation are clearly apparent in Figure 8. The device size is approximately 150 × 150 nm^2^ (Figure 8a). The thicknesses of the Al, Cu, and GeSe films are approximately 160, 40, and 38 nm, respectively. The Ge_0.2_Se_0.8_ film in the filament region appears crystalline owing to Cu^*z*+^ ion migration and Cu filament formation under SET conditions (Figure 8b), which is also investigated by fast Fourier transform (FFT) image. Amorphous Ge_0.2_Se_0.8_ film is shown at the without-filament region (Figure 8c). The *d* spacing in the Cu electrode and filament regions is found to be approximately 6.94 and 6.51 Å (or 6.54 and 7.02 Å), respectively (Figure 8d,e,f), which is larger than our previous reported *d* spacing of Cu (111) (*d* approximately 2.087Å) [33]. The *d* spacing of Se was reported to be 5, 3.7, and 2.9 Å that correspond to the (001), (100), and (101) planes, respectively [50]. Those values are also lower than that observed values in the TEM image (Figure 8b). On the other way, considering the interplanar spacings *d*_1_ and *d*_2_ of two overlapping crystals, the *d* spacing of parallel moire’ fringes will be increased [51]. Even though the Cu will be mixed in the Ge_0.2_Se_0.8_ in the filament region, however, the *d* spacing of Cu will be also increased, which may be overlapping of different Cu nanocrystal fringes or Cu and GeSe nanocrystal fringes. Therefore, a crystalline Cu (or Cu/GeSe mixture) nanofilament is observed. Further study is needed to understand clearly the lattice fringes in the filament. The Cu filament diameter (approximately 30 nm) in the Cu/Ge_0.2_Se_0.8_/W device is larger than that (approximately 11 nm) in the Cu/Ge_0.4_Se_0.6_/W device [20], owing to the less controlled migration of Cu ions in Ge_0.2_Se_0.8_ than in Ge_0.4_Se_0.6_, and as a result of the lower barrier for hole injection. All layers of W, Ge_0.2_Se_0.8_, Cu filament, and Cu are confirmed in EDX spectra (Figure 8g). A strong Cu signal is observed in the crystalline region due to the formation of Cu clusters or a Cu nanofilament within the Ge_0.2_Se_0.8_ solid electrolyte. There are two main diffusion mechanisms, one involving fast transport via interstices and a slow one by substitution. However, Cu^*z*+^ ions are highly mobile in chalcogenides and can also migrate rapidly as interstitial species until they encounter a vacancy, where substitution then occurs [52,53]. Therefore, the defects also play a role in the controlled formation and dissolution Cu filament through the solid electrolyte. 

**Figure 9:**
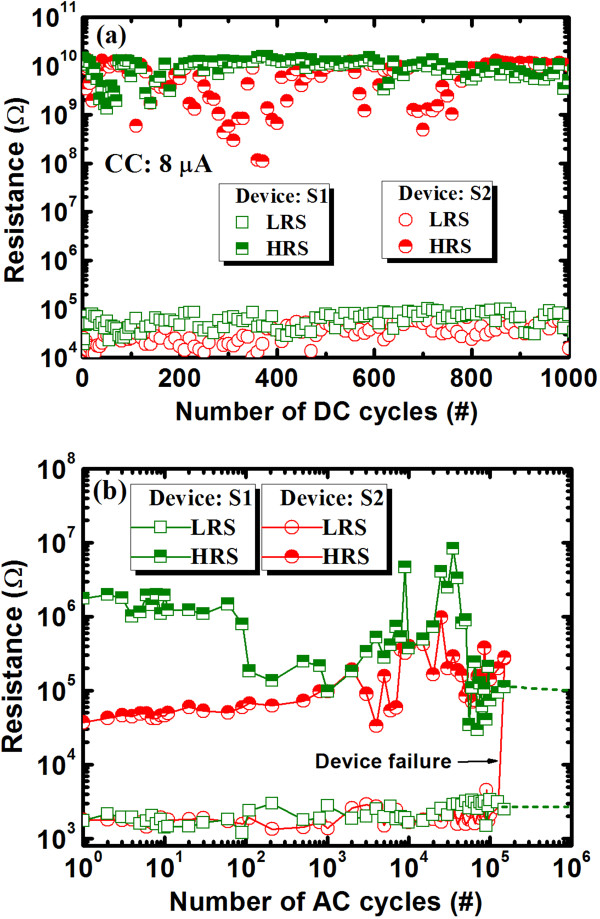
**Resistance states.** (**a**) The resistance states with DC cycles of the S1 and S2 devices. (**b**) AC endurances of approximately 10^5^ cycles are observed for both devices. However, the Ge_0.5_Se_0.5_ devices (with more Ge content) are more stable.

**Figure 10:**
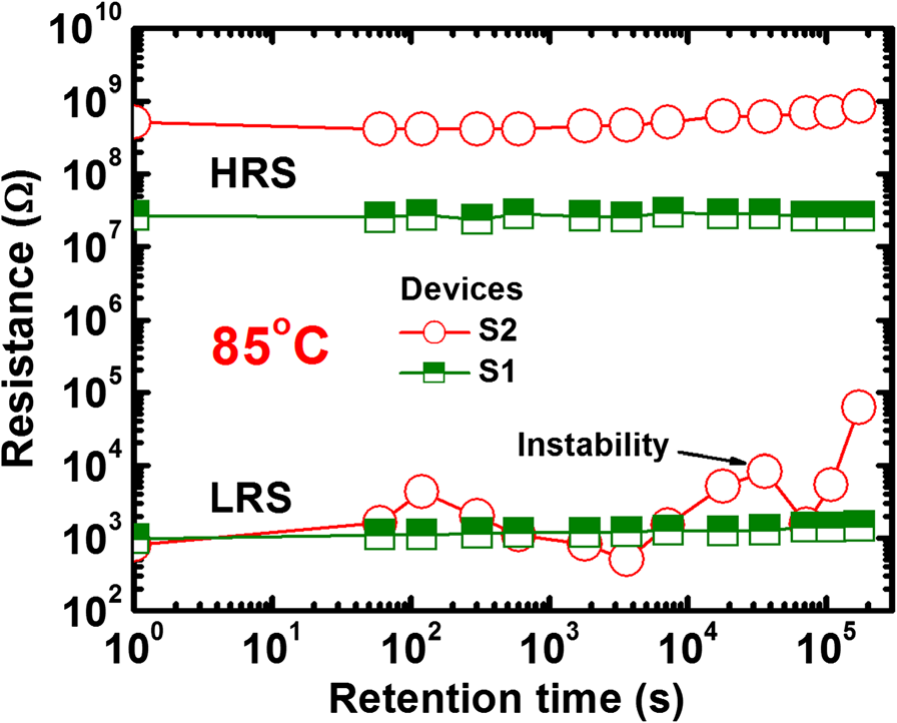
**Stable retention characteristics over** >**2 days at 85°C.** Obtained for the Ge_0.5_Se_0.5_device as compared to the Ge_0.2_Se_0.8_ device.

**Figure 11:**
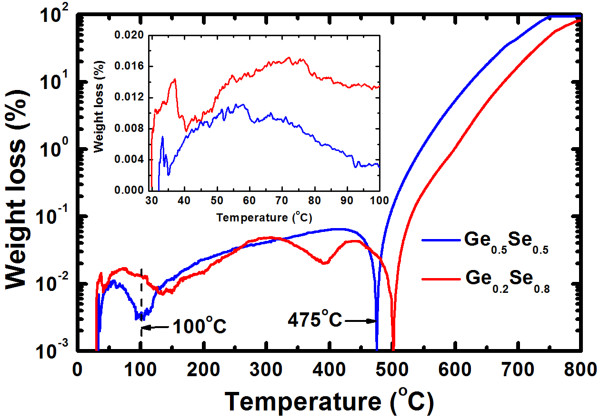
**Weight loss of the Ge**_**0**.**5**_**Se**_**0**.**5**_**and Ge**_**0**.**2**_**Se**_**0**.**8**_**switching materials.** The Ge_0.5_Se_0.5_ material shows lower loss below 100°C, indicating that a higher Ge content enhances thermal stability.

The structural flexibility of films with a higher Se content produces large variations in the HRS (Figure 9a) after few cycles, owing to the easier migration of Cu under the SET operation, which results in a higher RESET current (4.5 vs. 0.14 μA), as shown in Figure 2. However, a good DC endurance with a stable resistance state and a high resistance ratio of >10^4^ at a CC of 8 μA was observed in the high-Ge-content devices (S1). This results from the limited migration of Cu under the SET operation or from the controlled Cu nanofilament diameter under SET and RESET operations. The S1 memory devices show stable AC program/erase endurance, as shown in Figure 9b. The applied P/E current and pulse width were 500 μA and 0.5 ms, respectively. The programming and erasing voltages were set to +1.1 and −1.0 V, respectively, and the read voltage was 50 mV. The S1 devices were robust over >10^6^ P/E cycles, while the S2 devices showed inferior P/E cycles (they failed after 10^5^ cycles). Continuous P/E cycles can heat up the S2 devices, and hence, Se atoms may out-diffuse (or voids may be created in the Ge_0.2_Se_0.8_ solid electrolyte), resulting in device failure. Well-controlled Cu nanofilament formation and dissolution under the SET and RESET operations of the S1 devices has the advantage of long P/E cycles. Robust data retention characteristics at 85°C are shown in Figure 10. The P/E current was 300 μA. The S1 devices yield HRS (approximately 27.6 MΩ) and LRS (approximately 1 kΩ) both stable over >10^5^ s at 85°C, while the S2 devices yield stable HRS (0.5 GΩ) and unstable LRS (initially, 0.9 kΩ). This suggests an inferior thermal stability of Ge_0.2_Se_0.8_-based resistive switching memory devices. Figure 11 shows the weight loss of the switching materials as a function of temperature, obtained from thermo-galvanometric measurements. The temperature increases at a rate of 10°C/min. The flow rate of Ar was 200 ml/min. The weight loss of Ge_0.2_Se_0.8_ material is greater than Ge_0.5_Se_0.5_ material (1.3% vs. 0.3% at 100°C) because of the lower melting point of Se, as shown in the inset of Figure 11. This suggests that the Se atoms are reduced in the S2 devices at 85°C during data retention measurements, which destabilizes the LRS. Above 100°C, Ge_0.5_Se_0.5_ loses weight slowly and evaporates rapidly above 475°C, the melting point of Ge_0.5_Se_0.5_. Bruchhaus et al. [54] reported a thermal stability for Ge_0.4_Se_0.6_ up to approximately 450°C. Therefore, the high-Ge-content Ge_0.5_Se_0.5_ solid electrolyte is preferable for future nanoscale nonvolatile memory applications. 

**Figure 12:**
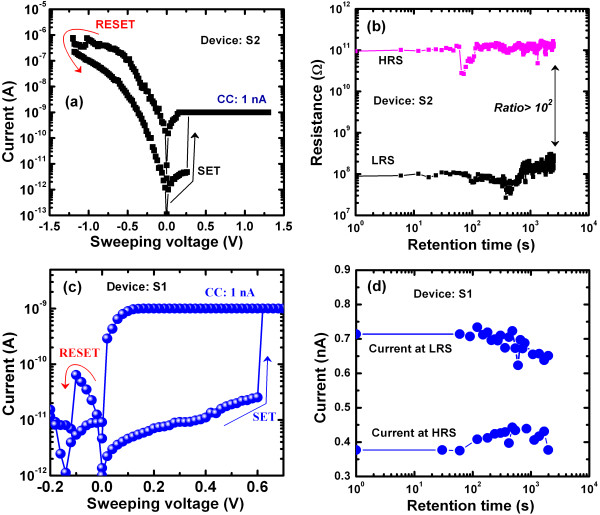
***I***-***V*hysteresis and data retention.** (**a**) Typical *I*-*V* hysteresis for the S2 device with a low CC of 1 nA. However, a high RESET current of 1 μA is observed owing to the faster migration of Cu^*z*+^ ions or a larger Cu filament diameter. (**b**) Good data retention is observed in the S2 devices for a CC of 1 nA. (**c**) Typical *I*-*V* hysteresis for the S1 devices. The observed low RESET current of approximately 64 pA is due to the controlled migration of Cu^*z*+^ ions. (**d**) Typical data retention characteristics at a small CC of 1 nA for a device size of 8 × 8 μm^2^.

This memory device can be also used for low-power operation. Figure 12a shows the *IV* hysteresis characteristics for a small CC of 1 nA in the S2 devices. However, the RESET current of approximately 1 μA is high, owing to the very strong migration of Cu in the Ge_0.2_Se_0.8_ solid electrolyte, even for a small CC of 1 nA, or to an overshoot effect [55]. This will make the scalability of Ge_0.2_Se_0.8_ devices more challenging. The higher RESET current makes the Cu filament diameter strong, thereby producing good data retention over >30 min, as shown in Figure 12b. On the other hand, the repeatable resistive switching memory characteristics with a small CC of 1 nA and a small RESET current of 64 pA are also observed in the S1 devices (Figure 12c). The lower RESET current is due to the thinner Cu nanofilament caused by the controlled migration of Cu^*z*+^ ions through the higher hole-injection barrier. These devices also show retention characteristics over a few minutes at a small CC of 1 nA (Figure 12d). However, the current at LRS decreases slowly after 100 s of retention time, which is probably a thinner filament diameter. The LRS and HRS have approximately 20 MΩ and approximately 30 GΩ, respectively, at a read voltage of +0.2 V (Figure 12c). The high *V*_SET_ and the large resistance ratio of >10^2^ will be useful for future logic memory design. However, lower SET and RESET powers are 0.61 nW and 6.4 pW, respectively. Considering the resistivity (*ρ*_filament_ approximately 200 μΩ cm [29]) of the Cu filament, the diameter for a small CC of 1 nA is approximately 0.25Å, indicating potential scalability beyond the atomic scale. By considering a low operation current of 1nA, an even denser memory of 1,300 Pbit/in^2^ could be designed in the future. 

## Conclusions

We investigated the superior and repeatable bipolar resistive switching memory characteristics of an Al/Cu/Ge_0.5_Se_0.5_/W structure, as compared to an Al/Cu/Ge_0.2_Se_0.8_/W structure, with a small operating voltage of ±1.5 V. The composition of the switching materials was confirmed using both EDX and XPS. We demonstrated a nanoscale memory device with a size of 150 × 150 nm^2^, as confirmed by HRTEM. This Al/Cu/Ge_0.5_Se_0.5_/W memory device has a higher *V*_SET_ of approximately 0.6 V, a stable endurance over >10^5^ cycles, and shows excellent data retention characteristics over a time of >10^5^ s at 85°C and a large resistance ratio of >10^4^. A lower barrier height for hole injection than for electron injection helps the propagation of the Cu^*z*+^ ions and the initiation of growth and dissolution at the Ge_0.5_Se_0.5_/W and Cu/Ge_0.5_Se_0.5_ interfaces, respectively. The migration of Cu^*z*+^ ions, via defects, into the Ge_*x*_Se_1 − *x*_ solid electrolyte explains the basic switching mechanism. The Cu nanofilament with a diameter of 30 nm is also observed by HRTEM under SET. The Al/Cu/Ge_0.5_Se_0.5_/W device can be operated with a current as low as 1 nA. Furthermore, the SET and RESET powers are small at 0.61 nW and 6.4 pW, respectively. This suggests that the solid electrolyte Ge_0.5_Se_0.5_, with a higher Ge content, in an Al/Cu/Ge_0.5_Se_0.5_/W structure paves the way to future atomic scale (<1 Å) nonvolatile memory applications.

## Competing interests

The authors declare that they have no competing interests.

## Authors’ contributions

SZR carried out the fabrication of the CBRAM device, measurement, and data analysis under the instruction of SM. AD and AP helped deposit the GeSe films under the instruction of SM. YHW helped partially fabricate the device under the instruction of SM. CSL and LBC provided the device fabrication facility. TCT performed the XPS measurement and analysis. WSC, HYL, FTC, and MJT contributed to the via structure design. All the authors contributed to the preparation and revision of the manuscript, approved it for publication, and read and approved the final manuscript.

## References

[B1] Rainer W Nanoelectronics and Information Technology: Advanced Electronic Materials and Novel Devices 2012 3 Wiley-VCH, Weinheim

[B2] Waser R Aono M Nanoionics-based resistive switching memories Nat Mater 2007 6 833 10.1038/nmat2023 17972938

[B3] Sawa A Resistive switching in transition metal oxides Mater Today 2008 11 28

[B4] Lee HY Chen PS Wang CC Maikap S Tzeng PJ Lin CH Lee LS Tsai MJ Low power switching of nonvolatile resistive memory using hafnium oxide Jpn J Appl Phys 2007 46 2175 10.1143/JJAP.46.2175

[B5] Afanas’ev VV Stesmans A Pantisano L Cimino S Adelmann C Goux L Chen YY Kittl JA Wouters D Jurczak M TiNx/HfO2interface dipole induced by oxygen scavenging Appl Phys Lett 2011 98 132901 10.1063/1.3570647

[B6] Sun X Li G Chen L Shi Z Zhang W Bipolar resistance switching characteristics with opposite polarity of Au/SrTiO3/Ti memory cells Nanoscale Res Lett 2011 6 599 10.1186/1556-276X-6-599 22107926 PMC3260421

[B7] Jeong DS Schroeder H Waser R Impedance spectroscopy of TiO2 thin films showing resistive switching Appl Phys Lett 2006 89 082909 10.1063/1.2336621

[B8] Kwon DH Kim KM Jang JH Jeon JM Lee MH Kim GH Li XS Park GS Lee B Han S Kim M Hwang CS Atomic structure of conducting nanofilaments in TiO2 resistive switching memory Nat Nanotechnol 2010 5 148 10.1038/nnano.2009.456 20081847

[B9] Lin CC Chang YP Lin HB Lin CH Effect of non-lattice oxygen on ZrO2-based resistive switching memory Nanoscale Res Lett 2012 7 187 10.1186/1556-276X-7-187 22416817 PMC3324381

[B10] Lin CY Wu CY Wu CY Lee TC Yang FL Hu C Tseng TY Effect of top electrode material on resistive switching properties of ZrO2 film memory devices IEEE Electron Device Lett 2007 28 366

[B11] Zhang T Zhang X Ding L Zhang W Study on resistance switching properties of Na0.5Bi0.5TiO3 thin films using impedance spectroscopy Nanoscale Res Lett 2009 4 1309 10.1007/s11671-009-9397-4 20628453 PMC2893894

[B12] Kim DC Seo S Ahn SE Suh DS Lee MJ Park BH Yoo IK Baek IG Kim HJ Yim EK Lee JE Park SO Kim HS Chung UI Moon JT Ryu BI Electrical observations of filamentary conductions for the resistive memory switching in NiO films Appl Phys Lett 2006 88 202102 10.1063/1.2204649

[B13] Panda D Dhar A Ray SK Nonvolatile and unipolar resistive switching characteristics of pulsed laser ablated NiO films J Appl Phys 2010 108 104513 10.1063/1.3514036

[B14] Chiu FC Li PW Chang WY Reliability characteristics and conduction mechanisms in resistive switching memory devices using ZnO thin films Nanoscale Res Lett 2012 7 178 10.1186/1556-276X-7-178 22401297 PMC3325859

[B15] Torrezan AC Strachan JP Medeiros-Ribeiro G Williams RS Sub-nanosecond switching of a tantalum oxide memristor Nanotechnology 2011 22 485203 10.1088/0957-4484/22/48/485203 22071289

[B16] Prakash A Maikap S Lai CS Lee HY Chen WS Chen FT Kao MJ Tsai MJ Improvement of uniformity of resistive switching parameters by selecting the electroformation polarity in IrOx/TaOx/WOx/W structure Jpn J Appl Phys 2012 51 04DD06

[B17] Wu Y Lee B Wong HSP Al2O3-based RRAM using atomic layer deposition (ALD) with 1-μA RESET current IEEE Electron Device Lett 2010 31 1449

[B18] Banerjee W Maikap S Lai CS Chen YY Tien TC Lee HY Chen WS Chen FT Kao MJ Tsai MJ Yang JR Formation polarity dependent improved resistive switching memory characteristics using nanoscale (1.3 nm) core-shell IrOx nano-dots Nanoscale Res Lett 2012 7 194 10.1186/1556-276X-7-194 22439604 PMC3338378

[B19] Kozicki MN Mitkova M Waser R Memory devices based on mass transport in solid electrolytes Nanotechnology. Volume 3 2008 Wiley-VCH, Weinheim

[B20] Rahaman SZ Maikap S Chiu HC Lin CH Wu TY Chen YS Tzeng PJ Chen F Kao MJ Tsai MJ Bipolar resistive switching memory using Cu metallic filament in Ge0.4Se0.6 solid-electrolyte Electrochem Solid-State Lett 2010 13 H159 10.1149/1.3339449

[B21] Yu S Wong HSP Compact modeling of conducting-bridge random-access memory (CBRAM) IEEE Trans Electron Dev 2011 58 1352

[B22] Jameson JR Gilbert N Koushan F Saenz J Wang J Hollmer S Kozicki MN One-dimensional model of the programming kinetics of conductive-bridge memory cells Appl Phys Lett 2011 99 063506 10.1063/1.3623485

[B23] Sakamoto T Lister K Banno N Hasegawa T Terabe K Aono M Electronic transport in Ta2O5 resistive switch Appl Phys Lett 2007 91 092110 10.1063/1.2777170

[B24] Schindler C Thermadam SCP Waser R Kozicki MN Bipolar and unipolar resistive switching in Cu-doped SiO2 IEEE Trans Electron Dev 2007 54 2762

[B25] Wang D Liu L Kim Y Huang Z Pantel D Hesse D Alexe M Fabrication and characterization of extended arrays of Ag2S/Ag nanodot resistive switches Appl Phys Lett 2011 98 243109 10.1063/1.3595944

[B26] Terabe K Hasegawa T Nakayama T Aono M Quantized conductance atomic switch Nature 2005 433 47 10.1038/nature03190 15635405

[B27] Liu Q Long S Lv H Wang W Niu J Huo Z Chen J Liu M Controllable growth of nanoscale conductive filaments in solid-electrolyte-based ReRAM by using a metal nanocrystal covered bottom electrode ACS Nano 2010 4 6162 10.1021/nn1017582 20853865

[B28] Li Y Long S Lv H Liu Q Wang Y Zhang S Lian W Wang M Zhang K Xie H Liu S Liu M Improvement of resistive switching characteristics in ZrO2 film by embedding a thin TiOx layer Nanotechnology 2011 22 254028 10.1088/0957-4484/22/25/254028 21572216

[B29] Rahaman SZ Maikap S Chen WS Lee HY Chen FT Tien TC Tsai MJ Impact of TaOx nanolayer at the GeSex/W interface on resistive switching memory performance and investigation of Cu nanofilament J Appl Phys 2012 111 063710 10.1063/1.3696972

[B30] Nagata T Haemori M Yamashita Y Yoshikawa H Iwashita Y Kobayashi K Chikyow T Bias application hard x-ray photoelectron spectroscopy study of forming process of Cu/HfO2/Pt resistive random access memory structure Appl Phys Lett 2011 99 223517 10.1063/1.3664781

[B31] Goux L Opsomer K Degraeve R Muller R Detavernier C Wouters DJ Jurczak M Altimime L Kittl JA Influence of the Cu-Te composition and microstructure on the resistive switching of Cu-Te/Al2O3/Si cells Appl Phys Lett 2011 99 053502 10.1063/1.3621835

[B32] Rahaman SZ Maikap S Tien TC Lee HY Chen WS Chen F Kao MJ Tsai MJ Excellent resistive memory characteristics and switching mechanism using a Ti nanolayer at the Cu/TaOxinterface Nanoscale Res Lett 2012 7 345 10.1186/1556-276X-7-345 22734564 PMC3436867

[B33] Rahaman SZ Maikap S Chen WS Lee HY Chen FT Kao MJ Tsai MJ Repeatable unipolar/bipolar resistive memory characteristics and switching mechanism using a Cu nanofilament in a GeOx film Appl Phys Lett 2012 101 073106 10.1063/1.4745783 PMC3436867 22734564

[B34] Peng S Zhuge F Chen X Zhu X Hu B Pan L Chen B Li RW Mechanism for resistive switching in an oxide-based electrochemical metallization memory Appl Phy Lett 2012 100 072101 10.1063/1.3683523

[B35] Yang Y Gao P Gaba S Chang T Pan X Lu W Observation of conducting filament growth in nanoscale resistive memories Nat Commun 2012 3 1737 10.1038/ncomms1737 22415823

[B36] Liu Q Sun J Lv H Long S Yin K Wan N Li Y Sun L Liu M Real-time observation on dynamic growth/dissolution of conductive filaments in oxide-electrolyte-based ReRAM Adv Mater 1844 2012 24 10.1002/adma.201104104 22407902

[B37] Kund M Beitel G Pinnow CU Röhr T Schumann J Symanczyk R Ufert KD Müller G Conductive bridging RAM (CBRAM): an emerging non-volatile memory technology scalable to sub 20 nm IEDM Tech Dig 2005 10.1109/IEDM.2005.1609463

[B38] Jeong DS Lim H Park GH Hwang CS Lee S Cheong BK Threshold resistive and capacitive switching behavior in binary amorphous GeSe J Appl Phys 2012 111 102807 10.1063/1.4714705

[B39] Ueno T Odajima A Study of photo-induced effect in obliquely-deposited amorphous Ge-Se films by XPS Jpn J Appl Phys 1980 19 L519 10.1143/JJAP.19.L519

[B40] Ueno T Odajima A X-ray photoelectron spectroscopy of Ag-and Cu-doped amorphous As2Se3and GeSe2 Jpn J Appl Phys 1982 21 230 10.1143/JJAP.21.230

[B41] Grubbs ME Deal M Nishi Y Clemens BM The effect of oxygen on the work function of tungsten gate electrodes in MOS devices IEEE Electron Dev Lett 2009 30 925

[B42] Anderson PA The work function of copper Phys Rev 1949 76 388 10.1103/PhysRev.76.388

[B43] Vegard L Die Konstitution der Mischkristalle und die Raumfüllung der Atome Zeitschrift für Physik 1921 5 17 10.1007/BF01349680

[B44] Cardarelli F Materials Handbook 2000 Springer, London

[B45] Jeong HY Kim SK Lee JY Choi SY Role of interface reaction on resistive switching of metal/amorphous TiO2/Al RRAM devices J Electrochem Soc 2011 158 H979 10.1149/1.3622295

[B46] Kim SY Lee JL Enhancement of optical properties in organic light emitting diodes using the Mg-Al alloy cathode and IrOx-coated indium tin oxide anode Appl Phys Lett 2006 88 112106 10.1063/1.2179108

[B47] Edwards TG Sen S Structure and relaxation in germanium selenide glasses and supercooled liquids: a Raman spectroscopic study J Phys Chem B 2011 115 4307 21446741 10.1021/jp202174x

[B48] Boolchand P Bresser WJ The structural origin of broken chemical order in GeSe2 glass Philosophical Magazine B: Physics of Condensed Matter; Statistical Mechanics, Electronic, Optical and Magnetic Properties 2000 80 1757

[B49] Bakr N Aziz M Hammam M Structural properties of GexSe1-x thin films prepared by semi-closed space technique Egypt J Sol 2000 23 45

[B50] Li X Li Y Li S Zhou W Chu H Chen W Li IL Tang Z Single crystalline trigonal selenium nanotubes and nanowires synthesized by sonochemical process Crystal Growth & Design 2005 5 911 10.1021/cg049681q

[B51] Zhou GW TEM investigation of interfaces during cuprous island growth Acta Mater 2009 57 4432 10.1016/j.actamat.2009.06.005

[B52] McHardy C Fitzgerald A Moir P Flynn M The dissolution of metals in amorphous chalcogenides and the effects of electron and ultraviolet radiation J Phys C: Solid State Phys 1987 20 4055 10.1088/0022-3719/20/26/010

[B53] Phillips JC Structural principles of alpha-AgI and related double salts J Electrochem Soc 1976 123 934 10.1149/1.2132971

[B54] Bruchhaus R Honal M Symanczyk R Kund M Selection of optimized materials for CBRAM based on HT-XRD and electrical test results J Electrochem Soc 2009 156 H729 10.1149/1.3160570

[B55] Kinoshita K Tsunoda K Sato Y Noshiro H Yagaki S Aoki M Sugiyama Y Reduction in the reset current in a resistive random access memory consisting of NiOx brought about by reducing a parasitic capacitance Appl Phy Lett 2008 93 033506 10.1063/1.2959065

